# The novel application of cordycepin in maintaining stem cell pluripotency and increasing iPS cell generation efficiency

**DOI:** 10.1038/s41598-020-59154-5

**Published:** 2020-02-10

**Authors:** Chie-Hong Wang, Cheng-Hsuan Chang, Tsung-Li Lin, Ru-Huei Fu, Yu-Chuen Huang, Shih-Yin Chen, Woei-Cherng Shyu, Shih-Ping Liu

**Affiliations:** 1 Graduate Institute of Biomedical Science, China Medical University, Taichung, Taiwan; 2 Department of Orthopedics, China Medical University Hospital, Taichung, Taiwan; 3 Center for Translational Medicine, China Medical University Hospital, Taichung, Taiwan; 4 Genetics Center, Department of Medical Research, China Medical University Hospital, Taichung, Taiwan; 5 School of Chinese Medicine, College of Chinese Medicine, China Medical University, Taichung, Taiwan; 6 Department of Social Work, Asia University, Taichung, Taiwan

**Keywords:** Induced pluripotent stem cells, Reprogramming

## Abstract

Maintaining the pluripotency of either embryonic stem (ES) cells or induced pluripotent stem (iPS) cells is a fundamental part of stem cell research. In this study, we reported that cordycepin promoted the expression of pluripotency markers in ES and iPS cells. ES cells treated with cordycepin demonstrated their potential for generating embryoid bodies and differentiating into all three germ layers. The expression levels of phospho-Jak2, phospho-Stat3, integrin αV, and integrin β5 were increased after cordycepin treatment. Furthermore, the protein expression levels of IL-6 family proteins (IL-6, IL-11, LIF, oncostatin M (OSM), ciliary neurotrophic factor (CNTF)), and epidermal growth factor (EGF) were also upregulated after cordycepin treatment, but were restored after co-treatment with a Jak2 inhibitor (AG490). The gene expression levels of Yamanaka factors were upregulated in mouse embryonic fibroblasts (MEFs) after cordycepin treatment. Moreover, the generation efficiencies of iPS cells were elevated after cordycepin treatment. We found that iPS cells generated after cordycepin treatment, not only expressed pluripotency markers, but also showed the ability of differentiating into neuron stem/progenitor cells. Taken together, we demonstrated that cordycepin maintained the pluripotency of stem cells via regulation of extracellular matrix (ECM) and Jak2/Stat3 signaling pathway and improved the generation efficiency of iPSCs.

## Introduction

There are two main types of pluripotent stem cells, embryonic stem (ES) cells and induced pluripotent stem (iPS) cells. ES cells are isolated from the inner cell mass of blastocysts, whereas iPS cells were first generated from the somatic cells by introducing Oct4, Sox2, c-Myc, and Klf4 genes^1^. Although both ES and iPS cells demonstrated the capacities to self-renew and differentiate into all cell types, iPS cells overcome the drawbacks of ES cells. iPS cells were speculated to be a powerful cell therapy material as they could be derived and generated from individuals, and used to generate cells for treatment and monitoring disease *in vitro* without any immune rejection and ethical concern.

Mouse leukemia inhibitory factor (LIF) was used in the culture medium of mouse ES and iPS cells to maintain their pluripotency by activating the Jak2/Stat3 pathway^2,3^. Cordycepin, also known as 3′-deoxyadenosine, is the major compound isolated from *Cordyceps sinensis* (a traditional Chinese medicine). It acts as a polyadenylation inhibitor and exhibits inhibitory effects on cell proliferation among several cancer types, including breast cancer^4^, prostate cancer^5^ and leukemia^6^. Interestingly, it was also found to protect against cerebral ischemia injury^7^. A previous study indicated that cordycepin prevented the TNF-α-induced inhibition of osteogenic differentiation of human adipose-derived mesenchymal stem cells^8^. Nevertheless, the role of cordycepin on maintaining the pluripotency of ES and iPS cells was still unclear.

To date, there were several strategies to enhance the reprogramming efficiency, including knockdown of p53 gene^9^, hypoxic conditions^10,11^, epigenetic modification^12^, regulation of microRNAs^13^ and addition of small molecular compounds^14,15^. In 2003, one group reported a near 100% reprogramming efficiency in mouse and human cells via OKSM transduction and Mbd3 depletion^16^. However, it is still important to develop an enhanced reprogramming strategy without altering the genome integrity. In this study, we evaluated the effects of cordycepin on generation of iPS cells and maintaining pluripotency in both ES and iPS cells. Our data indicated that cordycepin is capable of enhancing the iPS cell generation efficiency and maintaining the pluripotency of ES and iPS cells by activating Jak2/Stat3 signaling and the ECM pathway.

## Results

### Cordycepin maintained the pluripotency of embryonic stem cells and induced pluripotent stem cells

**Figure 1:**
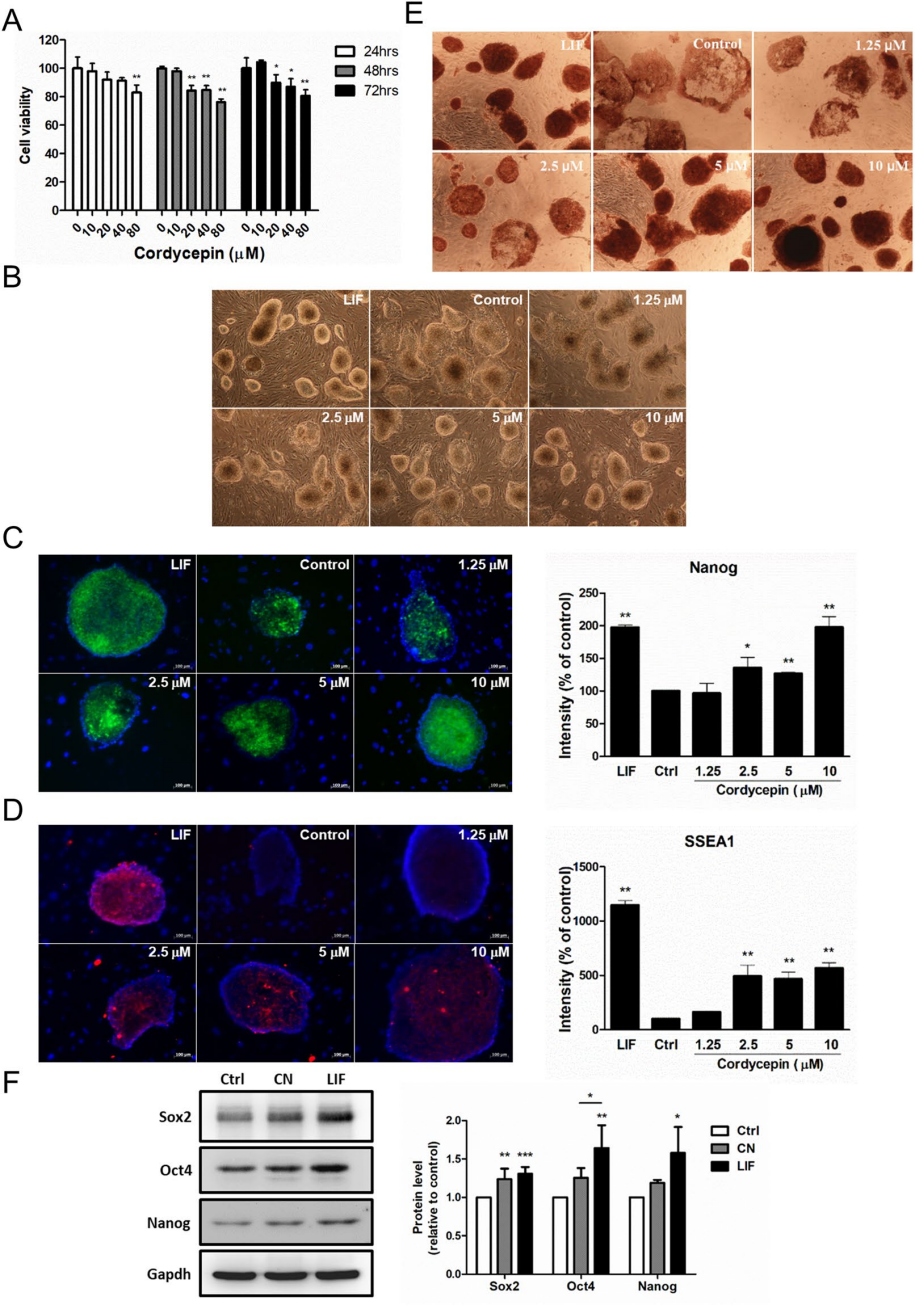
Cordycepin promoted the expression of pluripotent markers in mouse ES and iPS cells. (**A**) The cytotoxicity of cordycepin on mouse embryonic fibroblasts was assessed by MTT assay. (**B**) The phase contrast images of mouse ES cells which cultured in medium containing either LIF (1,000 units/ml) or different concentrations of cordycepin (0 to 10 μM) for 72 hours. Digital images were taken at a magnification of 100X. (**C**,**D**) Mouse ES cells were cultured in medium containing either LIF (1,000 units/ml) or different concentrations of cordycepin (0 to 10 μM) for 72 hours. The protein expression levels of Nanog and SSEA1 were assessed by immunofluorescent staining. Digital images were taken at a magnification of 200X (scale bar: 100 μm). (**E**) The activities of alkaline phosphatase were detected by immunocytochemistry staining in ES cells treated with cordycepin. (**F**) The protein expression levels of Sox2, Oct4 and Nanog in iPS cells treated with ctrl (LIF-negative stem cell medium), CN (cordycepin, 10 μM) or LIF medium (stem cell medium) for 72 hours. The data were collected from at least three independent experiments. Digital images were taken at a magnification of 100X. Bars represent mean and SD. Differences between the control group (Ctrl) and experimental groups (LIF and cordycepin) were evaluated by two-tailed Student’s t test or one-way ANOVA. *P < 0.05 indicates statistical significance (*P = 0.01–0.05; **P = 0.001–0.01).

Since cordycepin has been reported to inhibit cell growth among several cell types, we examined the viability of cordycepin-treated MEF cells by an MTT assay in a time- and dose-dependent manner. Our data indicated that cordycepin, at concentrations higher than 10 μM, decreased the viability of MEF cells during different time intervals (Fig. 1A). To minimize the interference raised by its inhibitory effect on cell viability, the cordycepin treatment was performed with a maximum dose of 10 μM. Next, we assessed whether cordycepin regulated the expression of pluripotent genes in ES cells as compared to the regular mouse LIF supplement (1,000 units/ml) after 72 hours treatment. The phase contrast images showed that mouse ES and iPS cells in control groups (without LIF and cordycepin) and low concentration cordycepin groups (1.25 μM to 5 μM) spontaneously differentiated (Fig. 1B and Supplementary Fig. S1A, respectively). Three pluripotent markers (i.e., Nanog, stage-specific embryonic antigen-1 (SSEA1), and alkaline phosphatase) were selected to evaluate the role of cordycepin in maintaining stem cell properties. Immunofluorescent staining data showed that treatment with 2.5 to 10 μM of cordycepin upregulated the expression of Nanog protein in ES cells. Furthermore, the effect of LIF on regulation of Nanog expression was mimicked by treatment with 10 μM of cordycepin (Fig. 1C). The expression of SSEA1 protein was about 10 times higher in LIF-treated ES cells compared to control group, whereas cordycepin treatment induced a five-fold increase of SSEA1 expression in ES cells compared to control group (Fig. 1D). In addition, we examined the protein expression levels of pluripotent genes in cordycepin-treated iPS cells. As shown in Supplementary Fig. S1B, the expression of Nanog protein was upregulated by cordycepin treatment in a dose-dependent manner. Cordycepin treatment induced a four- to nine-fold increase in the expression of Nanog in iPS cells compared to that in the control group. Although the SSEA1 protein expression levels increased after cordycepin treatment, the effects of cordycepin on SSEA1 expression were still less intense than those of LIF (Supplementary Fig. S1C). In addition, the result of immunocytochemistry staining indicated that cordycepin as well as LIF treatments increased the protein expression levels of alkaline phosphatase in both ES and iPS cells (Fig. 1E and Supplementary Fig. S1D, respectively). Furthermore, we isolated the embryonic fibroblasts from Oct4-GFP transgenic mice and reprogrammed these cells into iPS cells. As shown in Supplementary Fig. S1E, we found a four-fold increase in the expression levels of Oct4-GFP fusion protein, controlled by Oct4 promoter, in cordycepin-treated cells compared to that in the control group. Our data indicated that cordycepin exhibited regulatory effects on Oct4 expression, which were comparable to LIF treatment. The effect of cordycepin on maintaining stem cell pluripotency was also evaluated by incubating iPS cells with ctrl (LIF-negative stem cell medium), CN (cordycepin, 10 μM) or LIF medium (stem cell medium) for 72 hours. The protein expression levels of stem cell markers: Sox2, Oct4 and Nanog were detected by western blot (Fig. 1F). The results suggested that cordycepin maintain the protein expression levels of these stem cell markers, whereas the LIF treatment showed stronger effects.

**Figure 2:**
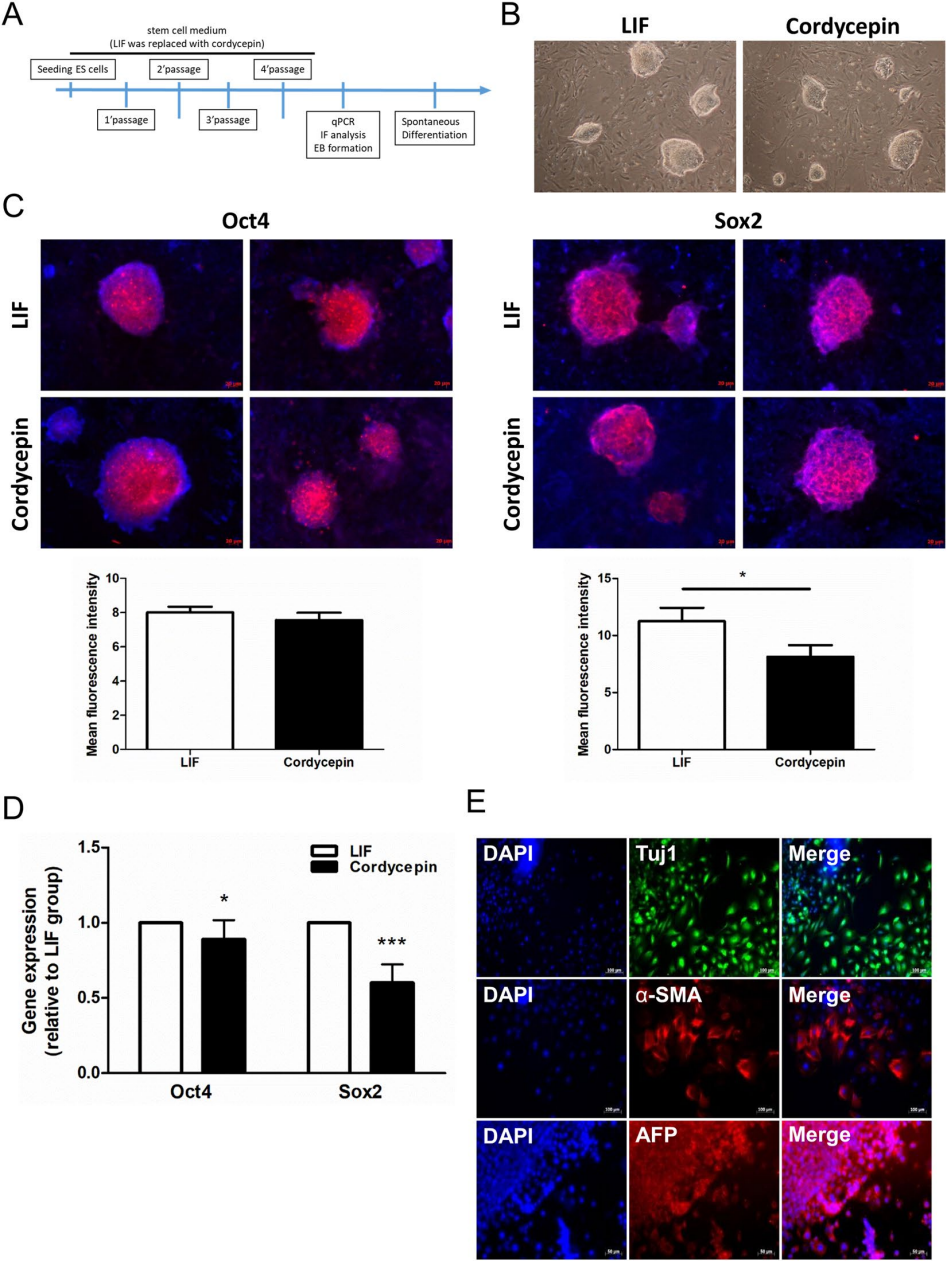
Cordycepin maintained the pluripotency of ES cells. (**A**) The strategy for long-term cordycepin treatment. Mouse ES cells were cultured in stem cell medium (without LIF) supplied with cordycepin (10 μM). Cells were routinely passaged every 3 to 4 days for 4 passages and then collected for further studies. (**B**) The morphology of mouse ES cell colonies incubating with LIF or cordycepin after 4 passages. The photos were documented by a phase-contrast microscope. (100X) (**C**) The expression levels of pluripotent genes (Oct4 and Sox2) in mouse ES cells cultured with LIF or cordycepin for four passages were assessed by immunofluorescent staining. Digital images were taken at a magnification of 100X (scale bar: 20 μm). (**D**) Real-time PCR assay was performed to determine the expression of pluripotent genes (Oct4, and Sox2) in mouse ES cells cultured with LIF or cordycepin for four passages. (**E**) These ES cells were then grown into embryoid bodies and seeded onto 0.1% gelatin-coated cell culture plate containing differentiation medium. The expression of markers specific for the three germ-layer in EB-outgrowth cells was visualized by immunofluorescent staining. Digital images were taken at a magnification of 200X (Tuj1 and α-SMA, scale bar: 100 μm) and 100X (AFP, scale bar: 50 μm). Bars represent mean and SD. Differences between the control group (LIF) and experimental groups (cordycepin) were evaluated by two-tailed Student’s t test. *P < 0.05 indicates statistical significance (*P = 0.01–0.05).

Next, we examined the effects of cordycepin on regulating mouse ES cell pluripotency after longer treatment. Mouse ES cells were cultured in stem cell media supplied with LIF or cordycepin (10 μM) for four passages and collected for further analysis (Fig. 2A). After incubated with cordycepin for four passages, these ES cell colonies contained tightly packed cells and exhibited round morphologies with well-defined sharp edges as well as cells incubated with LIF (Fig. 2B). The immunofluorescent staining data indicated that cordycepin treatment exhibited a comparable effect to those ES cells treated with LIF upon regulating the expression of Oct4 (Fig. 2C, left panel), whereas the expression of Sox2 showed a near 30% decrease in cordycepin group (Fig. 2C, right panel). Next, we examined the expression levels of pluripotent genes among this two groups The qPCR data indicated that there was no difference in the expression level of Oct4 between LIF and cordycepin groups, whereas the decreased expression levels of Sox2 in cordycepin group was observed (40%) (Fig. 2D). In addition, the effect of cordycepin on stem cell differentiation was determined by generating embryoid bodies (EBs) from ES cells. After incubation in a cordycepin-supplemented medium for four passages, embryoid bodies were generated. These embryoid bodies were seeded onto cell culture plates for expansion. The EB-outgrowth cells were then probed with fluorescent-dye conjugated antibodies against specific markers for the three germ layers (i.e., ectoderm: α-tubulin 3 (Tuj1), mesoderm: α-smooth muscle actin (α-SMA), and endoderm: alpha-fetoprotein (AFP)). Our data showed that cordycepin maintained the differentiation potential of ES cells, and that EB-outgrowth cells expressed specific markers for all three germ layers (Fig. 2E). Taken together, our data indicated that cordycepin maintained the pluripotency of both ES and iPS cells.

### Cordycepin maintained the pluripotency of ES cells by activating Jak2/STAT3 signaling

**Figure 3:**
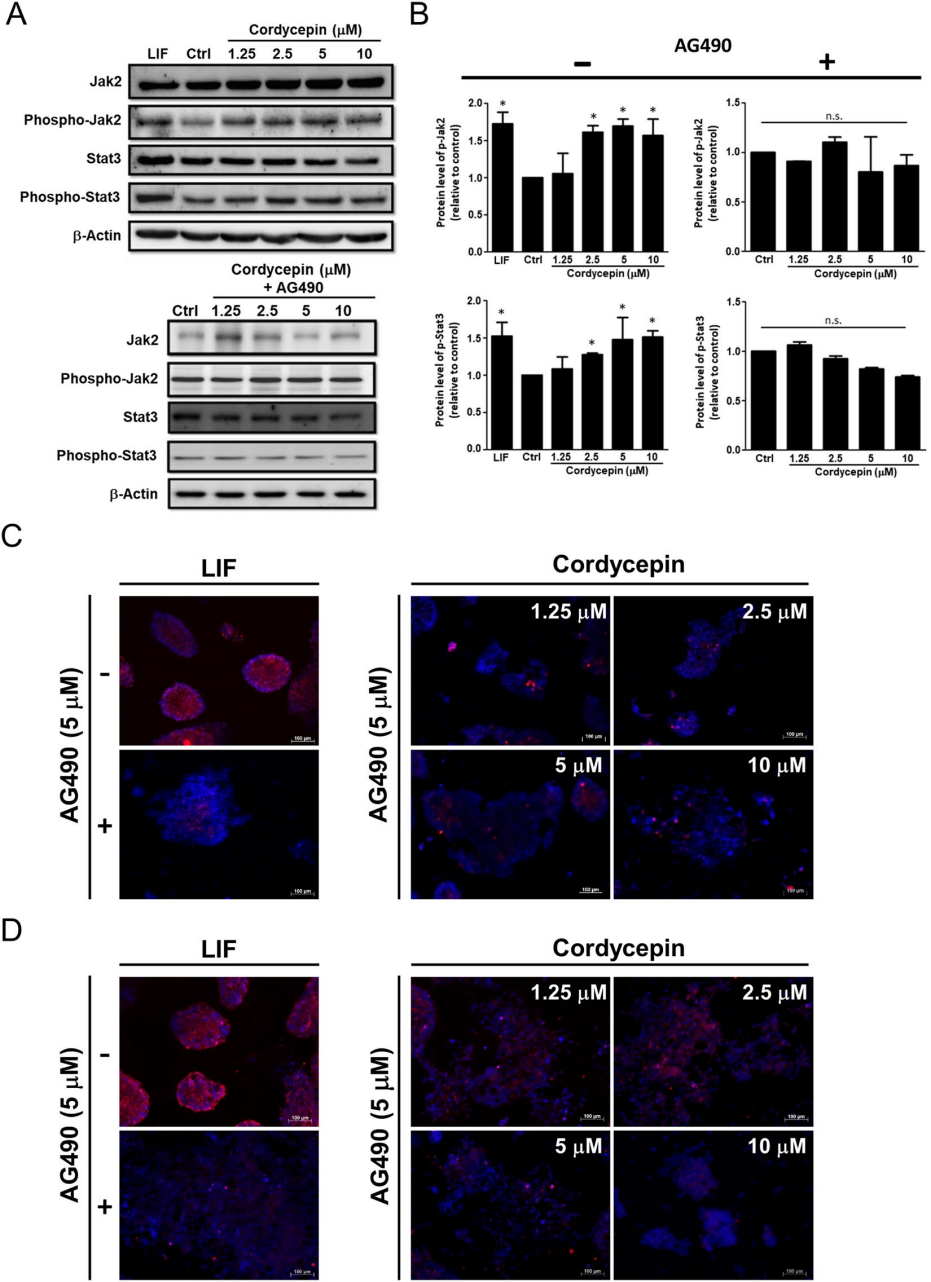
Cordycepin-mediated signaling pathway in mouse ES cells. (**A**,**B**) Western blot analyses of Jak2, Stat3 and their phosphorylated forms in mouse ES cells following either LIF (1000 units/ml) or cordycepin treatment (1.25, 2.5, 5, 10 μM). Jak2 inhibitor (AG490) was used to assess the effects of cordycepin on Jak2 and Stat3 activation. (**C**,**D**) Immunofluorescent staining results of Nanog and SSEA1 protein in cordycepin-treated mouse ES cells. AG490 was added to verify the effects of cordycepin on maintaining stem cell properties. Digital images were taken at a magnification of 200X (scale bar: 100 μm). Bars represent mean and SD. Differences between the control group (Ctrl) and experimental groups (LIF and cordycepin) were assessed by two-tailed Student’s t test. *P < 0.05 indicates statistical significance (*P = 0.01–0.05).

Next, we conducted microarray analysis to explore the genes regulated by cordycepin in ES cells. Numbers and percentages of significantly regulated genes with known biological functions are listed in Table 1. Several genes were regulated by cordycepin in ES cells, including the genes involved in signaling transduction, cell proliferation, metabolism, cell adhesion, and apoptosis. As indicated by previous studies, LIF activated the Jak2/Stat3 signaling pathway to maintain the pluripotency of mouse ES cells^17^. Interestingly, genes involved in the Jak-Stat signaling pathway were regulated by cordycepin (20 out of 46 genes were involved in Jak-Stat signaling pathway). We further examined the protein expression levels of Jak2, Stat3 and their phosphorylated forms. Western blotting data showed that the protein expression levels of phospho-Jak2 and phospho-Stat3 were elevated in ES cells treated with either LIF (1,000 units/ml) or cordycepin (2.5 to 10 μM). A specific Jak2 inhibitor, AG490, was used to verify the effect of cordycepin on Jak2/Stat3 activation. After co-treatment of cordycepin and AG490 (5 μM) for 72 hours, the expression of phospho-Jak2 and phospho-Stat3 in cordycepin-treated groups were strongly suppressed, whereas the expression levels of Jak2 or Stat3 remained unaffected or slightly decreased (Fig. 3A,B). Another potential mechanism was the signal transduction pathways derived from the interactions between the extracellular matrix (ECM) and its receptors (Table 1, 21 out of 37 genes were involved in ECM-receptor interaction)^18^. We found that cordycepin induced the expressions of integrin αV and β5 in both ES cells (Supplementary Fig. S2A) and iPS cells (Supplementary Fig. S2B). We also analyzed the expression profile of stem cell property-related genes (Supplementary Fig. S2C and Table S1)^19^. The results indicated that There were 9 genes (4%, group A) showed two-fold downregulation in cordycepin group as compared to LIF group, whereas there were 40 genes (16%, group F) showed two-fold upregulation in cordycepin group as compared to LIF group. In addition, there were 107 genes (42%, group E) showed one to two-fold upregulation in cordycepin group as compared to LIF group, and there were 94 genes (37%, group C) showed modest downregulation in cordycepin group as compared to LIF group. Collectively, these data indicated that cordycepin regulated the expression of stem cell property-related genes. Cordycepin enhanced the expression of stem cell property-related genes as compared to LIF (59%, group D, E and F), whereas it also downregulated the expression of several stem cell property-related genes (41% group A, B and C).

**Table 1:** Genes regulated by cordycepin in ES cells.

Function/Pathway	Cordycepin (10 μM)			
	Up^a^	Down^b^	No.	%
**Signal transduction**				
ECM-receptor interaction	4	17	21/37	56.76
Jak-Stat signaling pathway	6	14	20/46	43.48
Calcium signaling pathway	4	18	22/51	43.14
VEGF signaling pathway	1	3	4/12	33.33
PPAR signaling pathway	1	7	8/29	27.59
Insulin signaling pathway	12	4	16/59	27.12
TGF-beta signaling pathway	2	8	10/40	25.00
Wnt signaling pathway	4	9	13/52	25.00
MAPK signaling	4	18	22/97	22.68
**Cell proliferation**				
Cell communication	8	11	19/33	57.58
Cell cycle	6	9	15/67	22.39
**Metabolism**				
Amino acid metabolism	3	3	6/21	28.57
Lipid metabolism	7	7	14/69	20.29
**Cell adhesion**				
Cell adhesion molecules	9	18	27/55	49.09
Focal adhesion	5	26	31/78	39.74
Tight junction	9	8	17/51	33.33
**Apoptosis**				
Apoptosis	0	14	14/41	34.15

^a^Number of up-regulated genes.

^b^Number of down-regulated genes.

While previous studies showed that the expression of Nanog and SSEA1 were regulated by Stat3^20,21^, we also measured the protein expression levels of Nanog in cordycepin-treated ES cells with or without AG490 treatment. As shown in the left panel of Fig. 3C,D, the expression levels of Nanog and SSEA1 were increased after LIF treatment, whereas the addition of AG490 (5 μM) reversed these effects. In addition, our data showed that AG490 significantly suppressed the cordycepin-mediated increase in expressions of Nanog and SSEA1 (Fig. 3C,D, right panel) in ES cells. Taken together, these results demonstrated that cordycepin contributed to the maintenance of stem cell properties of ES cells potentially by activating the Jak2/Stat3 and ECM-receptor signaling pathway.

### Cordycepin induced the expression of IL-6 family proteins in ES cells

**Figure 4:**
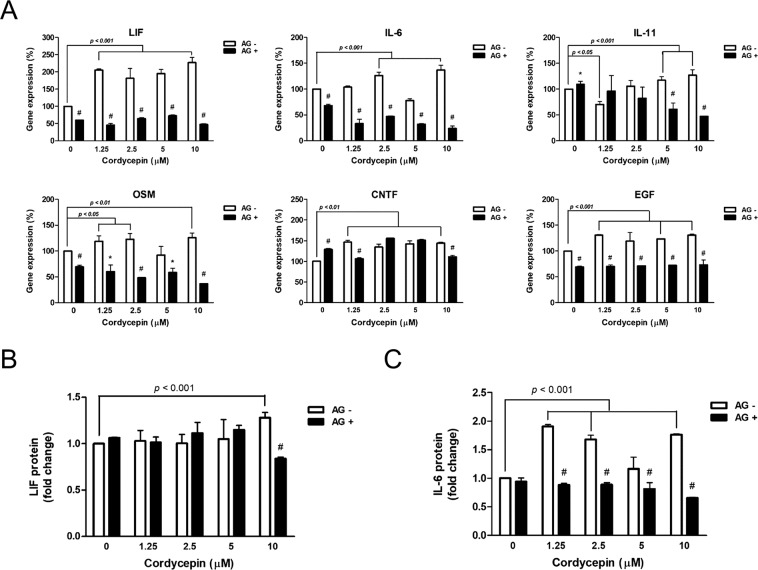
Cordycepin induced the expression of IL-6 family proteins and EGF in ES cells. (**A**) Real-time PCR assay was performed to determine the expression of LIF, IL-6, IL-11, oncostatin M (OSM), ciliary neurotrophic factor (CNTF), and epidermal growth factor (EGF) in cordycepin-treated mouse ES cells. AG490 was used to validate the role of cordycepin in promotion of expressions of LIF-receptor-related cytokines. (**B**,**C**) The cordycepin-mediated expressions of LIF and IL-6 in mouse ES cells were examined by ELISA. AG490 was used to evaluate the relationship between cordycepin and Jak2. Bars represent mean and SD. The statistical analysis was conducted using two-tailed Student’s t test (^#^p = 0.001–0.01 versus AG negative control).

Several interleukin-6 (IL-6) family proteins (e.g., LIF, IL-6, IL-11, oncostatin M (OSM), and ciliary neurotrophic factor (CNTF)) activate the Jak2/Stat3 signaling pathway via the common receptor subunit gp130^22^. In this study, we examined the expression levels of these IL-6 family proteins by real-time PCR to validate our hypothesis that cordycepin activated the Jak2/Stat3 signaling pathway in ES cells by inducing the expression of cytokines. Our data showed that, at the concentrations of 1.25 μM and above, cordycepin induced an almost two-fold increase in the expression of LIF compared to that in the control, whereas other IL-6 family cytokines (IL-6, IL-11, OSM, and CNTF) only showed a modest increase in their expressions (<1.5-fold). We also measured the gene expression level of epidermal growth factor (EGF) in cordycepin-treated ES cells, since EGF has previously been reported to induce the activation of STAT3^23^. Results indicated that cordycepin upregulated the expression of EGF in ES cells (Fig. 4A). Furthermore, the expressions of LIF, IL-6, OSM, and EGF but not IL-11 and CNTF were inhibited by AG490 in control groups, and the effects of cordycepin on the expression of LIF, IL-6, OSM, and EGF were also reversed by AG490. As shown in Fig. 4B, the protein expression levels of LIF were modestly increased upon cordycepin treatment and were suppressed by AG490. The expression levels of IL-6 protein were enhanced upon cordycepin stimulation, and co-treatment with AG490 significantly inhibited the effects of cordycepin on IL-6 expression (Fig. 4C). Taken together, these results provided the evidence that cordycepin induced the expressions of IL-6 family cytokines by activating the gp130/Jak2/Stat3 signaling pathway in ES cells, and these cytokines may activate gp130/jak2/Stat3 signaling via autocrine or paracrine mechanisms.

### Cordycepin promoted the generation of iPS cells

**Figure 5:**
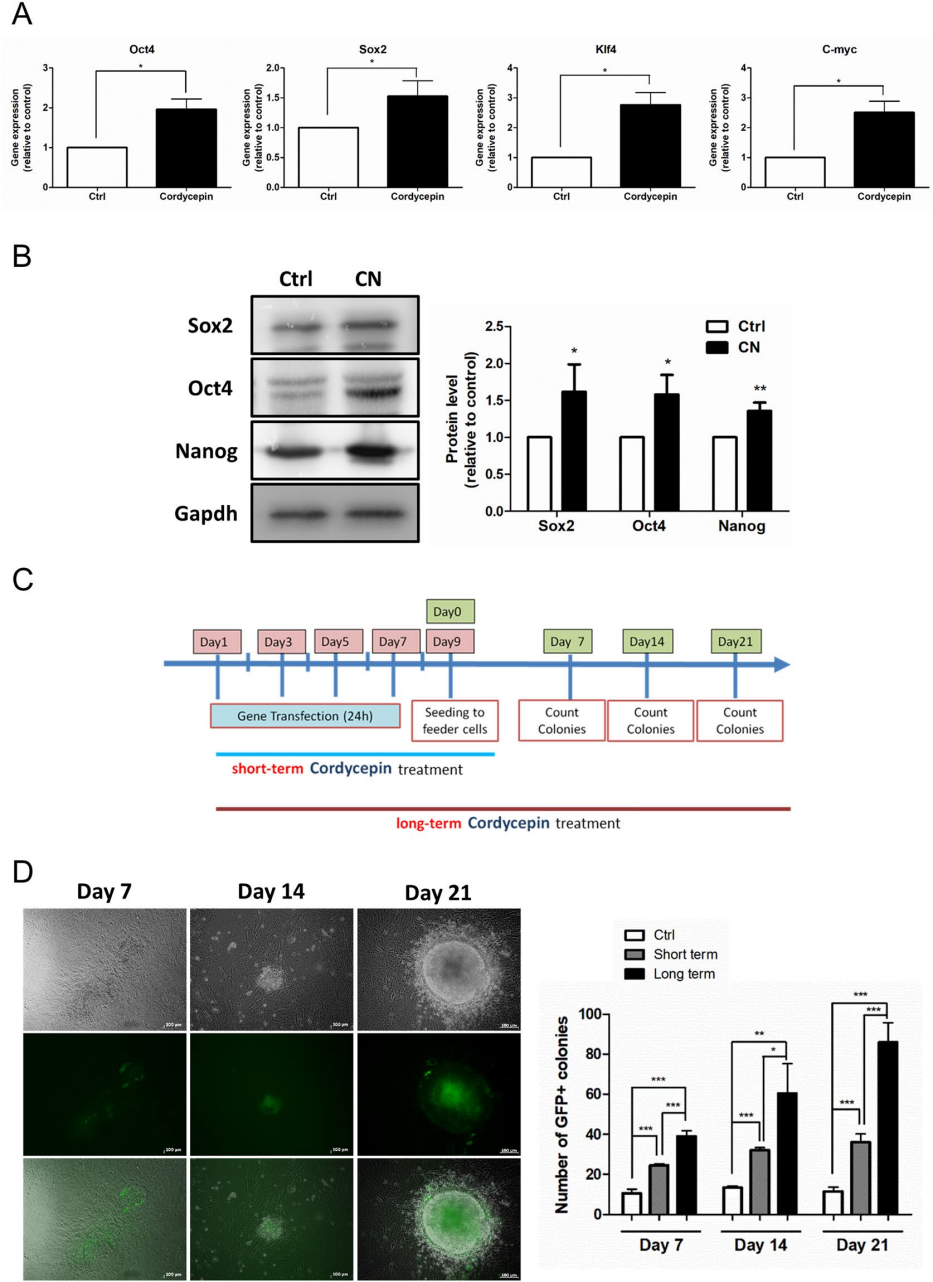
Cordycepin promoted the generation of iPS cells. (**A**) Real-time PCR assay was conducted to examine the expression levels of reprogramming factors in cordycepin-treated MEFs. (**B**) The protein expression levels of Sox2, Oct4 and Nanog in cordycepin-treated MEFs. MEFs were treated with cordycepin (10 μM) and the medium were refreshed every 48 hours for 120 hours. (**C**) The strategy for reprogramming MEFs (isolated from Oct4-GFP transgenic mice) into iPS cells with cordycepin treatment. (**D**) The embryonic fibroblasts isolated from Oct4-GFP transgenic mice were reprogrammed into iPS cells following the reprogramming strategy. The representative images of iPS colonies at day 7, 14 and 21 were documented by a fluorescence microscope. The number of iPS colonies between different groups was calculated at different time points. The data were collected at least from three independent experiments. Bars represent mean and SD. Differences between groups were assessed by one-way ANOVA with post Newman-Keuls Multiple Comparison Test. *P < 0.05 indicates statistical significance (*P = 0.01–0.05; **P = 0.001–0.01; ***P < 0.001).

Several reprogramming methods have been developed for generating iPS cells from somatic cells; however, the efficiency of reprogramming protocol was still a challenge for induced pluripotent stem cell research^24^. In 2008, one study reported that small molecule weight compounds (e.g., valproic acid) can improve the reprogramming efficiency^25^. Since our previous data indicated that cordycepin enhanced the expression of pluripotent genes in both ES and iPS cells, we investigated the possibility that cordycepin could also promote the generation of iPS cells. MEFs were treated with cordycepin at a concentration of 10 μM for 24 hours and then harvested for analysis. We found that cordycepin promoted the expression of Yamanaka factors (Oct4, Sox2, Klf4, and C-myc) in MEFs (Fig. 5A). The protein expression levels of Sox2, Oct4 and Nanog were also examined after cordycepin treatment (120 hours). As data showed in Fig. 5B, cordycepin upregulated the protein expression of Sox2, Oct4 and Nanog. We also explored the reprogramming efficiency in MEFs derived from Oct4-GFP transgenic mice following the protocol presented in Fig. 5C. The iPSC colonies were counted at day 7, 14, and 21 after seeding onto feeder cells. As shown in Fig. 5D, the number of iPSC colonies (GFP positive colonies) was increased in cordycepin groups as compared to control groups at different time intervals. In addition, prolonged cordycepin treatment significantly enhanced the reprogramming efficiency of cells compared to the control and groups subjected to short-term treatment of cordycepin. Taken together, our data indicated that cordycepin promoted the expression of Yamanaka factors and enhanced the reprogramming efficiency in MEFs.

### iPS-cordycepin cells exhibited the ability of self-renewal and differentiation

**Figure 6:**
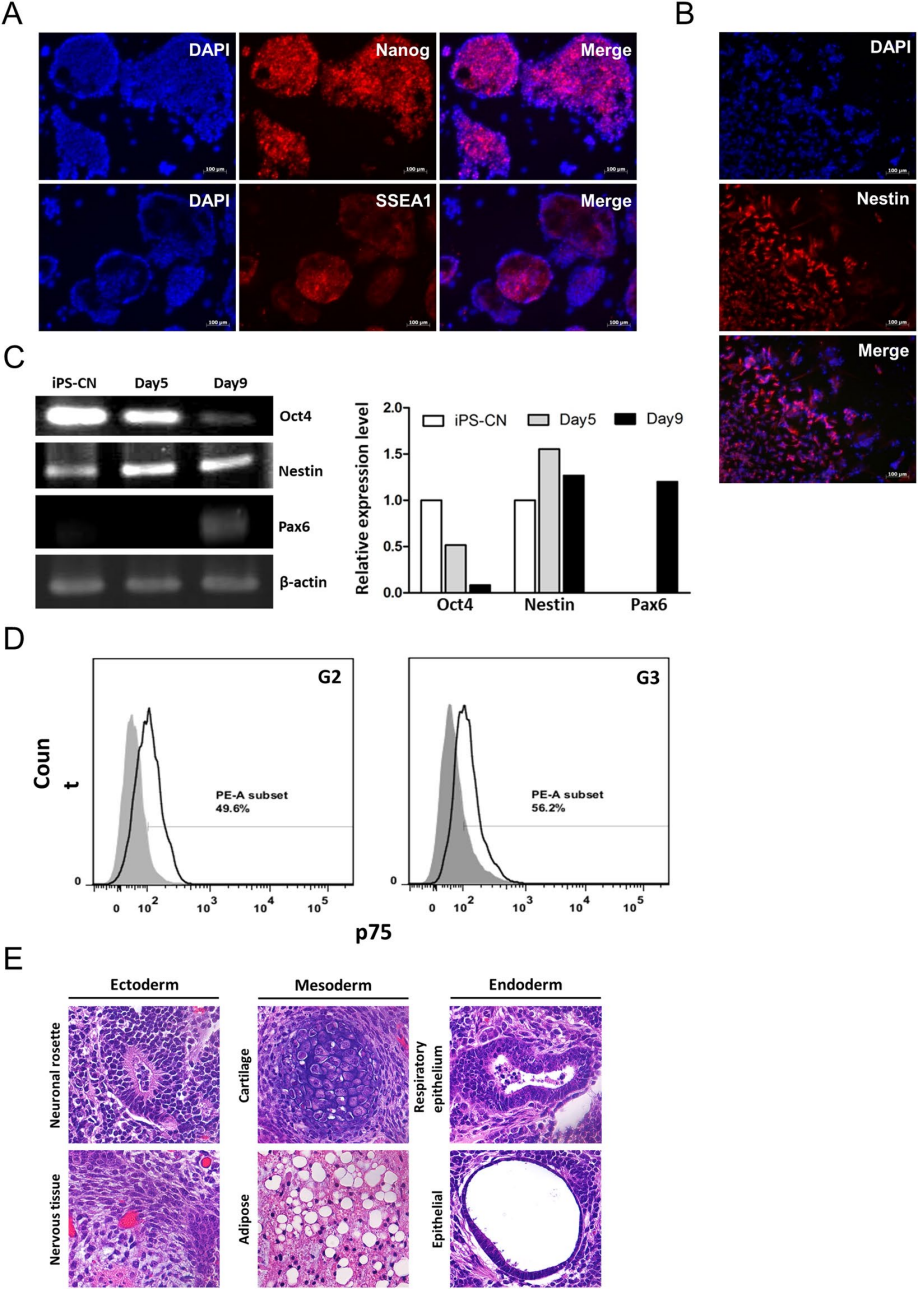
Cordycepin maintained the stem cell properties of iPS-cordycepin cells. iPS-cordycepin (iPS-CN) cells were generated by gene transfection, combined with long-term cordycepin treatment in MEFs derived from Oct4-GFP transgenic mice. These cells were maintained in medium containing 10 μM cordycepin. (**A**) Naong (upper panel) and SSEA1 (lower panel) were examined by immunofluorescent staining in iPS-CN cells. Digital images were taken at a magnification of 200X (scale bar: 100 μm). (**B**) The differentiation potential of iPS-CN cells was evaluated by differentiating into neural stem/progenitor cells. The protein expression level of Nestin was examined by immunofluorescent staining. Digital images were taken at a magnification of 200X (scale bar: 100 μm). (**C**) The gene expression levels of stem cell marker (Oct4) and neural stem/progenitor cell markers (Nestin and Pax6) were assessed by reverse transcriptase PCR during different time intervals during neural stem cell differentiation. (**D**) Flow cytometric analysis of the expression of neurotrophin receptor (p75) in different generations of neural stem cells. (**E**) The effects of cordycepin on maintaining stem cell pluripotency were evaluated by *in vivo* teratoma formation assay. HE stain results indicated that the iPS-CN cells possessed the ability to differentiate into all the 3-germ layer cells. Digital images were taken at a magnification of 200X.

iPS-cordycepin (iPS-CN) cells were generated by gene transfection, combined with long-term cordycepin treatment in MEFs derived from Oct4-GFP transgenic mice. Similar to ES cells, iPS-CN cells formed adherent, sharp-edged, tightly packed colonies when co-cultured with feeder cells (Supplementary Fig. S3A). Immunofluorescent staining data showed upregulated expressions of two pluripotency markers (Nanog and SSEA1) in iPS-CN cells (Fig. 6A). The differentiation potential of iPS-CN cells was evaluated by differentiating into neural stem/progenitor cells following the protocol described in Supplementary Fig. S3B. We found that iPS-CN cells exhibited the ability to grow embryoid bodies in suspension culture environment. To explore the differentiation potential of iPS-CN cells, these EBs were seeded onto culture dishes, and the outgrow cells were stained for specific markers for three germ layers. As shown in Supplementary Fig. S3C, these EB outgrow cells expressed markers specific for three germ layers, which indicated that iPS-CN cells possessed the ability to differentiate into all cell types. After expansion, cells were collected and stained for neural stem/progenitor cell marker (e.g., Nestin). Our data showed that these differentiated cells were positive for Nestin staining (Fig. 6B). We also compared the gene expression levels of Oct4 (pluripotent gene), and Nestin and Pax6 (markers of neural stem/progenitor cells) during different time intervals upon differentiation (Fig. 6C). The results indicated that the expression of Oct4 was downregulated, whereas, the expressions of Nestin and Pax9 were upregulated during differentiation. Moreover, we compared the expression levels of neurotrophin receptor (p75), another specific marker for neuron stem/progenitor cells in different generations of differentiated cells (G2: cells collected at day 3 during the expansion phase, G3: cells collected at day 6 during expansion phase). As shown in Fig. 6D, 49.6% of the p75-positive cells were in G2 phase and 56.2% in G3 phase. To further evaluate the effects of cordycepin on maintaining stem cell pluripotency, we performed the *in vivo* teratoma formation assay in immune-compromised mice as this assay was generally considered as a gold-standard assay to evaluate the stem cell pluripotency^26^. The teratoma was removed at Day 18 after transplantation, and the results of histological examination of the teratoma derived from iPS-CN cells upon subcutaneous transplantation revealed that iPS-CN cells spontaneously differentiate into cell types of the three germ layers (ectoderm: neuronal rosette and nervous tissue, mesoderm: cartilage and adipose tissue, and endoderm: respiratory epithelium and epithelial) (Fig. 6E). Taken together, these data indicated that iPS-CN cells exhibited self-renewal and differentiation abilities comparable to other iPS cells.

## Discussion

In this study, we found that cordycepin not only contributed to the maintenance of pluripotency and promoted the reprogramming efficiency, but also upregulated the IL-6 expression in mouse ES cells (Fig. 4C). The role of IL-6 in promoting iPS generation has been reported previously^27^. Moreover, it is well-established that the Jak/Stat signal is activated by IL-6 family proteins with homodimers of gp130 or heterodimers of gp130 and LIF receptor^28^. The activation of Stat3 was critical for the maintenance of pluripotency in ES cells^3^. Taken together, these reports indicated that IL-6/Jak/Stat3 signaling increased the generation efficiency of iPS cells and enhanced the maintenance of stem cell properties. Our data showed that cordycepin promoted the expression of IL-6 in mouse ES cells, similar to the findings of a previous study that reported that cordycepin also upregulated the expression of IL-6 in human peripheral blood mononuclear cells^29^. However, some studies have reported that cordycepin suppressed the expression of IL-6 in lipopolysaccharides-treated macrophages^30,31^, which indicated that the regulatory effects of cordycepin on IL-6 expression could vary with different cell types. In 2017, a study reported that cordycepin impaired the expressions of IL-8 and VCAM-1 but not IL-6 via repressing NF-κB signaling^32^. Their data suggested that there was a different regulatory pathway for regulating IL-6 expression in mesenchymal stem cells (MSCs). Additionally, the expression levels of IL-10, IL-6, IL-8, IL-1β and TNF-α were up-regulated in cordycepin-treated peripheral blood mononuclear cells (PBMCs)^29^. Moreover, a previous study found that different cell types showed different responses after cordycepin treatment. They reported that cordycepin reduced β-catenin expression in leukemia cells but not solid cancer cells^33^. Since the role of cordycepin in stem cells was still unclear, we examined the effects of cordycepin on stem cells in this study.

Although the inhibitory effects of cordycepin on mouse fibroblasts (NIH3T3) has been reported, we found that the low dose of cordycepin (10 μM) did not affect the cell proliferation of mouse embryonic fibroblasts. Interestingly, they also found that cordycepin inhibited mTOR signaling^34^. Meanwhile, other study indicated that LIF suppressed mTOR signaling to maintain the self-renewal and pluripotency of mouse ESCs^35^. Collectively, these findings suggested that cordycepin treatment induced different responses in different cell lines, and cordycepin maintained the pluripotency of mouse ESCs and iPSCs might through inhibiting mTOR signaling.

We examined the effects of cordycepin on promoting reprogramming efficiency in MEF cells isolated from Oct4-GFP transgenic mice. The results demonstrated that even short-term cordycepin treatment exhibited the ability to promote the generation of iPS cells (Fig. 5D). We speculated that this observation could be explained by the autocrine or paracrine mechanism of IL-6^36^. The treatment of cordycepin during gene transfection may activate the IL-6 signaling and the cells continued to express IL-6 during the reprogramming period, which, in turn, activated the Jak2/Stat3 signaling.

We observed an upregulation of LIF mRNAs in cordycepin-treated ES cells but not the protein levels of LIF. Therefore, we speculated that cordycepin regulated the expression of LIF mainly through the transcriptional regulatory mechanism (Fig. 4A,B). The expression of LIF can be regulated at the mRNA levels under hypoxic conditions, inflammatory stress, or stimulation by estrogen, TGF-β, and p53^37^. However, the translational regulation of LIF is still unclear. Furthermore, our data indicated that cordycepin significantly upregulated the expression of IL-6 but did not affect LIF expression in mouse ES cells (Fig. 4B,C). Taken together, our data indicated that cordycepin exhibits the potential for the maintenance of stem cell properties and promoting the reprogramming efficiency of iPS cells via activation of the gp130/Jak2/Stat3 signaling pathway.

## Materials and Methods

### Isolation of mouse embryonic fibroblasts and preparation of mitotic inactivated MEFs

Mouse embryonic fibroblasts (MEFs) were collected according to previous report^15^. Briefly, cells were isolated from 13.5-day-old C57BL/6 mice embryos (Taiwan National Laboratory Animal Center, Taipei, Taiwan) retrieved by Cesarean section. After removing the internal organs, legs, and heads, the remaining embryo parts were digested with trypsin-EDTA (GIBCO BRL; Grand Island, NY, USA) and cultured in Dulbecco’s modified Eagle’s/High glucose medium (GIBCO BRL) supplied with 10% heat-inactivated fetal bovine serum (GIBCO BRL), penicillin (100 U/ml; GIBCO BRL)), streptomycin (100 μg/ml; GIBCO BRL)), non-essential amino acids (0.1 mM; GIBCO BRL), and L-glutamine (2 mM; GIBCO BRL)) in a humidified incubator (37 °C) with 5% CO_2_. MEFs were treated with Mitomycin C (2.5 hours, 10 μg/ml; Roche, Basel, Switzerland) for the generation of MEF feeder cells. All experimental protocols were approved by the Institutional Animal Care and Use Committee of China Medical University.

### Cell culture of mouse ES and iPS cells

Mouse ES and iPS cells (Bioresource Collection and Research Center, Hsinchu, Taiwan) were co-cultured with MEF feeder cells in 0.1% gelatin-coated (Sigma-Aldrich, Saint Louis, USA) cell culture plates supplied with stem cell medium (DMEM/High glucose containing 15% FBS (Hyclone, Logan, UT, USA), non-essential amino acids (0.1 mM), L-glutamine (2 mM), β-mercaptoethanol (0.1 mM; GIBCO BRL), and LIF (1,000 units/ml; Millipore, Darmstadt, Germany)) in a humidified incubator (37 °C) with 5% CO_2_. To evaluate the effects of cordycepin on maintaining stem cell pluripotency, LIF was substituted by cordycepin in the stem cell medium.

### Cordycepin treatment and cell viability assay

Cordycepin (Sigma-Aldrich) was dissolved in DMSO (Sigma-Aldrich) and stored at −20 °C. The cytotoxicity of cordycepin in MEFs was assessed following previously described MTT procedures^15^. Briefly, cells were treated with different concentrations of cordycepin (DMSO: 0.1%) for different time interval before four-hour incubation with medium containing MTT (3-(4,5-dimethylthiazol-2-yl)−2,5-diphenyltetrazolium bromide; Sigma-Aldrich). After incubation, the purple crystal (formazan) was dissolve in DMSO and the absorbance was read at 540 nm with a standard plate reader.

### Immunofluorescence and alkaline phosphatase staining

The stem cells were cultured in stem cell medium supplemented with LIF or cordycepin for 72 hours, and then collected for immunofluorescent staining, alkaline phosphatase staining and western blot analysis. Immunofluorescent staining was performed according to the protocol described in a previous study^15^ using anti-Nanog (Novus, Saint Charles, MO, USA), anti-stage specific embryonic antigen 1 (SSEA1; Millipore, Billerica, MA, USA), anti-alpha smooth muscle actin (alpha-SMA), anti-neuron-specific class III beta-tubulin (Tuj1 antibody clone; both Chemicon, Temecula, CA, USA) monoclonal antibodies, and anti-alpha fetpreotin (AFP; Abcam, Cambridge, MA, USA) polyclonal antibodies. Alkaline phosphatase staining was performed using alkaline phosphatase substrate kits (Vector Lab, Burlingame, CA, USA) according to manufacturer’s protocol. Results were collected from three independent experiments and the fluorescent intensity was measured by Image J software.

### Embryoid body formation and spontaneous differentiation

Embryoid body (EB) formation and spontaneous differentiation experiments were performed according to previous report^15^. Briefly, ES cells were cultured with cordycepin for 4 passages before seeding onto Ultra Low Cluster Plate (Corning, New York, USA) supplied with EB formation medium (DMEM/High glucose with 15% FBS, 0.1 mM β-mercaptoethanol, 2 mM L-glutamine and 1% NEAA) for 72 hours. The EBs were seeded into 0.1% gelatin-coated cell culture plates and incubated with differentiation medium (DMEM/High glucose with 15% FBS, 0.1 mM β-mercaptoethanol, 2 mM L-glutamine, 1% NEAA, penicillin (100 U/ml) and streptomycin (100 μg/ml).

### Jak2 inhibitor and Western blot

The Jak2 specific inhibitor, AG490 (Sigma-Aldrich), was dissolved in ethanol at a concentration of 5 mM and stored at −20 °C. The working concentration of AG490 in culture medium is 5 μM. Cell lysates were collected by using RIPA buffer containing proteinase and phosphatase inhibitors. The proteins were separated by SDS-PAGE (Sigma-Aldrich) and transferred to a PVDF membrane (Millipore). After blocking by 5% non-fat milk in TBST, the membrane was incubated with appropriately diluted primary antibodies. Rabbit anti-Jak2 (Cell Signaling Technology, Danvers, MA, USA), mouse anti-phospho-Jak2 (Tyr1007/1008) (Cell Signaling), rabbit anti-Stat3 (BD Pharmingen, San Diego, CA, USA), rabbit anti-phospho-Stat3 (Ser727) (Cell Signaling), rabbit anti-EGFR (GeneTex, Irvine, CA, USA), rabbit anti-phospho-EGFR (Tyr1068) (GeneTex), rabbit anti-ERK (GeneTex), rabbit anti-phospho-ERK (Thr202/Tyr2004) antibodies (Invitrogen), rabbit anti-Sox2 (GeneTex), rabbit anti-Oct4 (GeneTex), rabbit anti-Nanog (Biorbyt) and mouse anti-beta-actin (Millipore) were used in this research. The enhanced chemilumescent (ECL) western blotting substrate kit (Thermo) was used to stain the membranes and signals were obtained using UVP BioSpectrum Imaging System (Upland, CA, USA).

### RNA extraction, Real-Time PCR and reverse transcription-PCR

TRIzol (Invitrogen, Carlsbad, CA, USA) was used to extract total RNA from cells. RNA concentrations were determined by spectrophotometry. Complementary DNA was produced from RNA using SuperScript III Reverse Transcriptase Kits (Invitrogen). Real-time PCR was performed as previously described to determine the gene expression levels of octamer binding transcription factor 4 (Oct4), sex determining region Y box 2 (Sox2), klf4, C-myc, Integrin αV, Integrin β5, LIF, IL-6, IL-11, oncostatin M, EGF, CNTF^38^. The gene expression levels of Oct4, Nestin and Pax6 in iPS-CN cells were examined using One-Step RT-PCR kit (GeneDireX, Las Vegas, Nevada, USA). The detail information of primer sequences was listed in Supplementary Table S2.

### Microarray analysis

iPS cells were treated with cordycepin (10 μM) or LIF for 24 hours, and then the total RNA was extracted by using TRIzol reagent. The gene expression profile was detected and analyzed by using Mouse G3 Whole Genome Oligo 8 × 60 K Microarray, Micro Scanner System and GeneSpring GX software (Agilent, Santa Clara, California, USA).

### Determining the generation efficiency of iPS cells

We isolated the MEF cells from Oct4-GFP transgenic mice (Jackson Lab, Bar Harbor, Maine, USA) and introduced these cells with plasmids expressing Oct4, Klf4, Sox2 and c-Myc (pCX-OKS-2A and pCX-cMyc; Addgene, Cambridge, MA, USA) once every two days (4 times total). From day 1 to day 4, cells were cultured in Dulbecco’s modified Eagle’s/High glucose medium supplied with 10% heat-inactivated fetal bovine serum, penicillin (100 U/ml), streptomycin (100 μg/ml), non-essential amino acids (0.1 mM), and L-glutamine (2 mM) in a humidified incubator at 37 °C with 5% CO2. The culture medium was replaced with stem cell medium at day 5. The transfected cells were sub-cultured onto MEF feeder cells, and the culture media were refreshed every two days to day 21. Cordycepin was added into the culture medium as the following conditions (Short term: cells were only treated with Cordycepin-containing medium before seeding to the feeder cells; Long term: cells were treated with Cordycepin-containing medium before and after seeding to the feeder cells. The GFP-positive iPS colonies were counted at day 7, 14 and 21 with a fluorescent microscope.

### *In vivo* teratoma formation assay

The *in vivo* teratoma formation assay was performed according to a previous study^26^. Briefly, the iPS-CN cells were incubated with stem cell medium supplied with cordycepin (10 μM) for 6 passages. These iPS-CN cells well suspended in phosphate buffered saline with matrigel (30%) and subcutaneously injected into the back of 5-week-old CAnN.Cg-Foxn1nu/CrlNarl mice (1 × 10^6^ cells in 200 μl volume). The mice were sacrificed at Day 18 after transplantation. The teratoma were collected and stained with hematoxylin and eosin. The cell types of the three germ layers were examined by a pathologist.

### Differentiate iPS-CN cells into Neural stem/precursor cells

The iPS cells generated by co-incubated with cordycepin (long-term) were named iPS-CN cells. The iPS-CN cells were transferred to Ultra-Low attached culture dishes and incubated in DMEM/High glucose containing 15% FBS, non-essential amino acids (0.1 mM), L-glutamine (2 mM), β-mercaptoethanol (0.1 mM) to generate embryoid bodies. After 4 days, the embryoid bodies were transferred to regular cell culture dishes containing ITS-FN medium (DMEM/F12 (GIBCO BRL), L-glutamine (2 mM), penicillin (100 U/ml), streptomycin (100 μg/ml), 1% ITS-G media supplement (GIBCO BRL) and 5 μg/ml Fibronectin (GIBCO BRL)) for neural stem/precursor cell selection (4 days). After selection, the cells were transferred to poly-L-ornithine (GIBCO BRL)/fibronectin-coated dishes containing medium with N-2 medium (DMEM/F12, L-glutamine (2 mM), penicillin (100 U/ml), streptomycin (100 μg/ml), 1% N-2 media supplement (GIBCO BRL)) supplied with basic fibroblast growth factor (bFGF; GIBCO BRL) for neural stem/precursor cell expansion. The final neural stem/precursor cells were collected for the further analysis.

### Flow cytometry analysis

The neural stem/precursor cells were collected and incubated with fluorescent dye (PE) conjugated anti-p75 antibody (Santa Cruz Biotechnology, Dallas, Texas, USA). The signal was detected by BD LSR II flow cytometer and the results were analyzed by FlowJo software (Ashland, Oregon, USA).

### Study approval

The animal studies were conducted in accordance with a protocol approved by the Institutional Animal Care and Utilization Committee of China Medical University, Kaohsiung, Taiwan. All experiments were performed in accordance with relevant guidelines and regulations of Taiwan (R.O.C.).

### Statistical analysis

Results were presented as mean ± SD. Student’s t-test and one-way ANOVA were used to evaluate the differences. All analyses were performed using GraphPad Prism 5.0 software (La Jolla, CA, USA) with a statistical significance level of *p* < 0.05.

## Supplementary information

Supplementary information.

## Data Availability

All data generated or analyzed during this study are included in this published article.
