# Supplementary material for: The novel application of cordycepin in maintaining stem cell pluripotency and increasing iPS cell generation efficiency
Source: Sci Rep. 2020 Feb 10;10:2187. doi: 10.1038/s41598-020-59154-5 (PMC7010772; doi:10.1038/s41598-020-59154-5)

**The novel application of cordycepin in maintaining stem cell pluripotency and increasing iPS cell generation efficiency**

Chie-Hong Wang^1^, Cheng-Hsuan Chang^1^, Tsung-Li Lin^2^, Ru-Huei Fu^1,3^, Yu-Chuen Huang^4,5^, Shih-Yin Chen^4,5^, Woei-Cherng Shyu^1,3^, Shih-Ping Liu^1,3,6*^

^1^Graduate Institute of Biomedical Science, China Medical University, Taichung, Taiwan

^2^Department of Orthopedics, China Medical University Hospital, Taichung, Taiwan,

^3^Center for Translational Medicine, China Medical University Hospital, Taichung, Taiwan

^4^Genetics Center, Department of Medical Research, China Medical University Hospital, Taichung, Taiwan

^5^School of Chinese Medicine, College of Chinese Medicine, China Medical University, Taichung, Taiwan

^6^Department of Social Work, Asia University, Taichung, Taiwan

**Supplementary Figure Legends**

**Supplementary Figure S1. Cordycepin treatment maintained the pluripotency of mouse iPS cells.**

(A) The phase contrast images of mouse iPS cells which cultured in medium containing either LIF (1,000 units/ml) or different concentrations of cordycepin (0 to 10 μM) for 72 hours. Digital images were taken at a magnification of 100x. (B and C) Mouse iPS cells were cultured in medium containing either LIF (1,000 units/ml) or different concentrations of cordycepin (0 to 10 μM). The protein expression levels of Nanog and SSEA1 were examined by immunofluorescent staining. Digital images were taken at a magnification of 200x. (D) The activities of alkaline phosphatase were detected by immunocytochemistry staining in iPS cells treated with cordycepin. (E) The embryonic fibroblasts isolated from Oct4-GFP transgenic mice were reprogrammed into iPS cells. These iPS cells were cultured in medium containing either LIF (1,000 units/ml) or different concentrations of cordycepin (0 to 10 μM). The expression levels of Oct4-GFP protein were examined by immunofluorescent staining. Digital images were taken at a magnification of 200x. Bars represent mean and SD. Differences were assessed by two-tailed Student’s t test.*P < 0.05 indicates statistical significance (*P = 0.01-0.05; ** P = 0.001- 0.01; ***P < 0.001).

**Supplementary Figure S2. The underlying mechanism of cordycepin-mediated maintenance of pluripotency.**

(A) Real-time PCR assay was performed to determine the expressions of integrin αV and β5 in cordycepin-treated mouse ES cells. (B) Real-time PCR assay was performed to determine the expressions of integrin αV and β5 in cordycepin-treated mouse iPS cells. (C) Whole genome analysis was performed to explore the effects of cordycepin on stem cell property-related genes. The gene expression levels was normalized to LIF-treated sample. Bars represent mean and SD. The statistical analysis was conducted using two-tailed Student’s t test (^#^p = 0.001-0.01, ** P = 0.001- 0.01; ***P < 0.001).

**Supplementary Figure S3. The neuron differentiation strategy and spontaneous differentiation abilities of iPS-CN cells.**

(A) Left panel: The phase contrast image of iPS-CN cells. Right panel: The expression of Oct4-GFP was documented by a fluorescent microscope. (B) The strategy for differentiating iPS-CN cells into neural stem cells (C) The expressions of specific markers for three germ-layer in EB-outgrowth cells generated by iPS-CN cells as visualized by immunofluorescent staining. Digital images were taken at a magnification of 200x (scale bar: 100 μm).

**Supplementary Figures**

**Supplmentary Figure S1.**

**
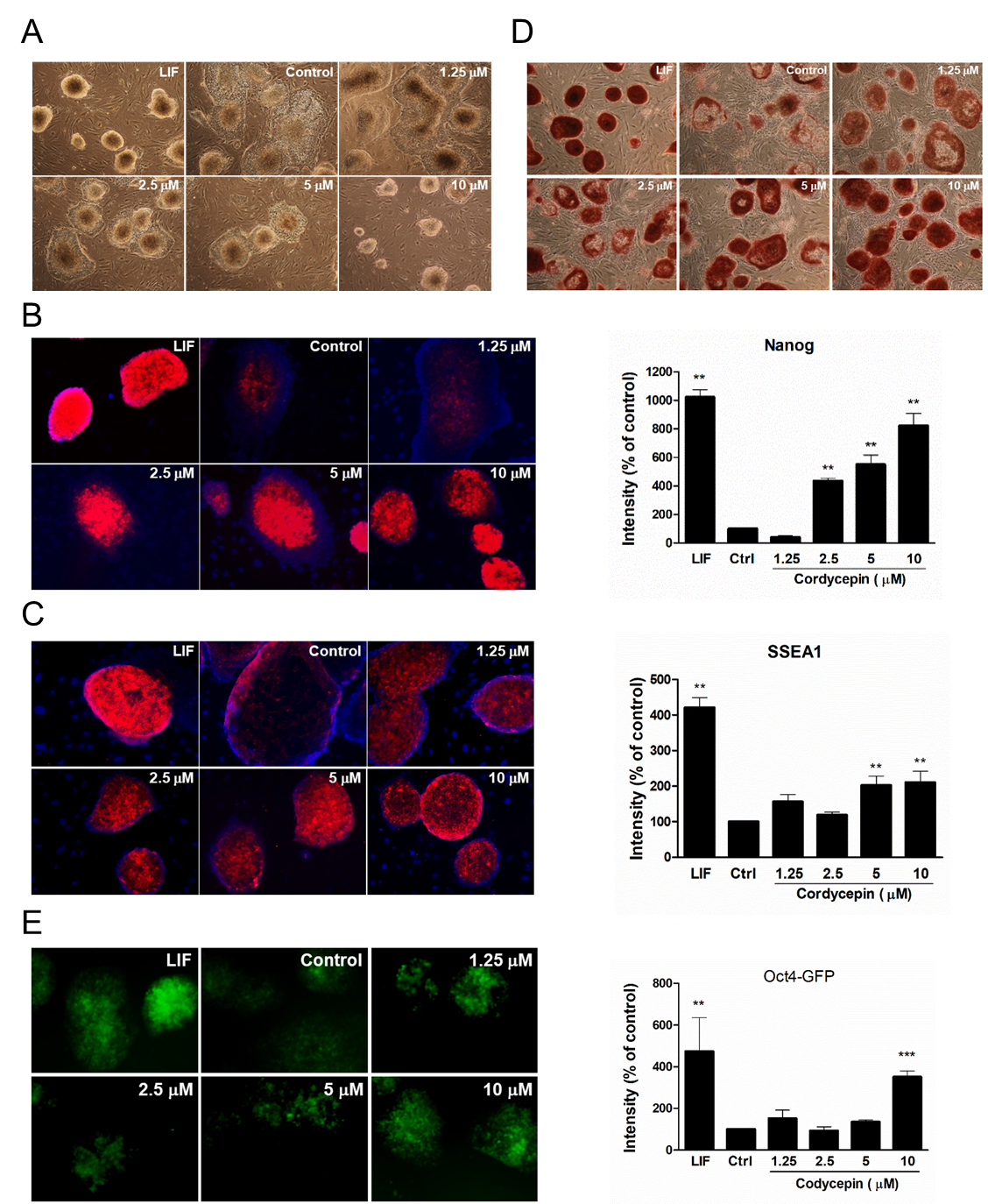
**

**Supplmentary Figure S2.**

**
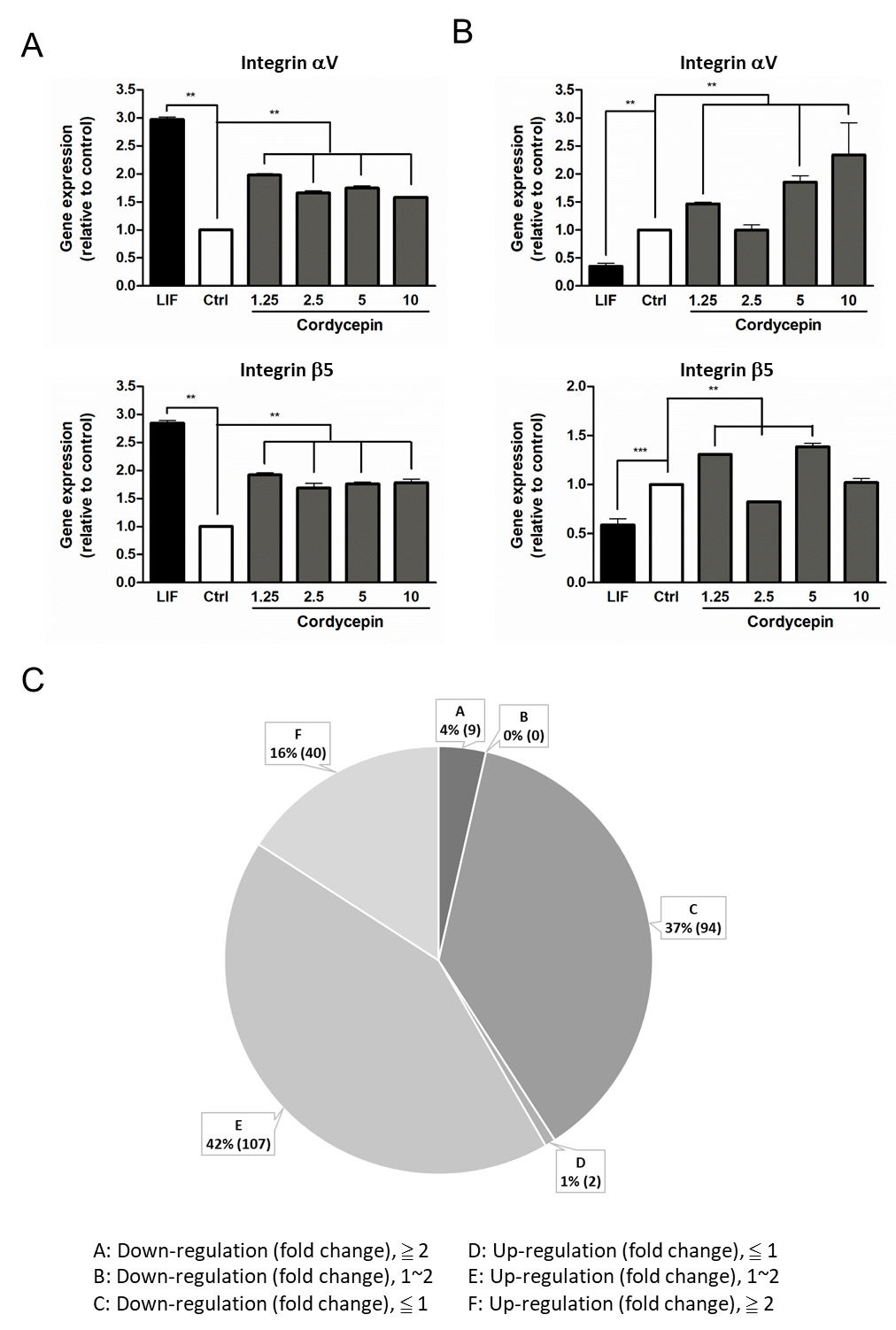
**

**Supplmentary Figure S3.**

**
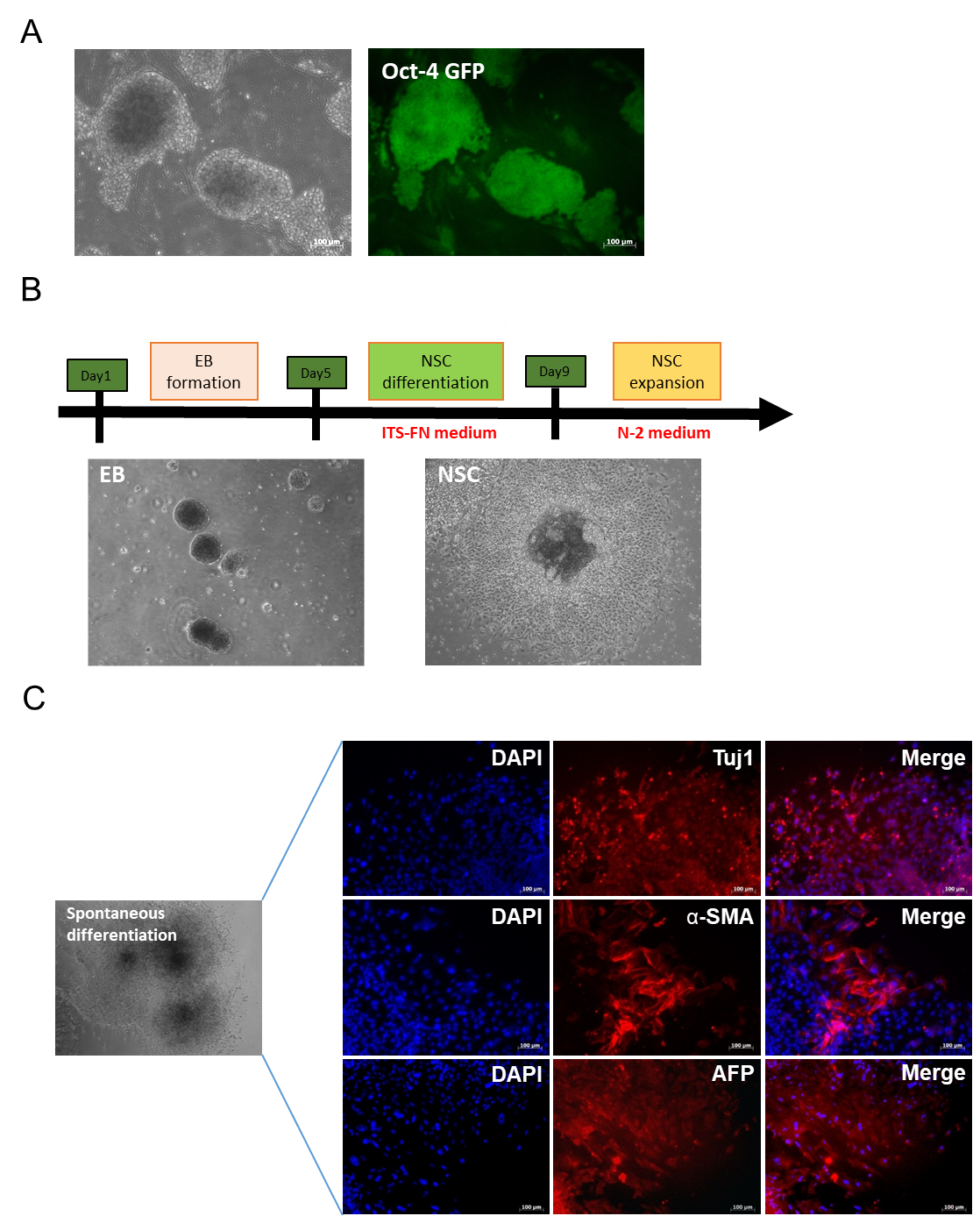
**

**Supplmentary Tables**

**Supplmentary Table S1. The lists of stem cell property-related genes.**

| **A** | **B** | **C** | | | | | **D** | **E** | | | | | | **F** | |
| --- | --- | --- | --- | --- | --- | --- | --- | --- | --- | --- | --- | --- | --- | --- | --- |
| NUP93 |  | RFC3 | GMPS | TET1 | ELMO1 | NR6A1 | MRPS27 | SKP2 | SFRS7 | RSL1D1 | PSIP1 | NUFIP1 | ACVR2B | BUB1 | PACSIN1 |
| DCP2 |  | TRIM24 | DNA2 | TMEM48 | SMEK1 | WDR12 | MTA3 | PPP1CC | RRM2 | POU5F1 | PDCD2L | PELI1 | ZWILCH | SPC25 | SMC4 |
| DDX21 |  | MCM7 | PTCH1 | POU2F1 | PPAT | RCL1 |  | HIC2 | CHEK2 | ALPL | HOOK1 | SH3GL3 | CDC25C | MND1 | E2F5 |
| POLR1B |  | OVOL2 | CAMKV | EXO1 | WDR3 | SNRPN |  | RAPGEF5 | SNX10 | BIRC5 | EXOSC3 | EZR | NAMPT | STRBP | CXADR |
| RRP1B |  | SFRS3 | HELLS | C1QBP | SET | SNX5 |  | ZMYND8 | BUB1B | MCM6 | DSCC1 | MLF1IP | TMEM180 | CCDC58 | APOE |
| HMGA1 |  | CDC25A | TLE3 | DDX18 | PLEKHA7 | WDR74 |  | UBE2C | ABCC4 | MATR3 | PWWP2A | RAB11FIP4 | PLCH1 | CKS2 | NUSAP1 |
| SLC5A6 |  | SNRPF | TAF4B | DDX11 | PHC1 | DNMT3B |  | GTSE1 | ASF1A | TFAM | TPX2 | SNRPD1 | RBM47 | CCNB2 | PPL |
| NOLC1 |  | PARP1 | FKBP4 | MCM5 | PATZ1 | MDN1 |  | WEE1 | CDC6 | MLLT4 | POLE2 | TEX9 |  | GCNT2 | MTF2 |
| E2F8 |  | RNF138 | SUPT16H | FBL | PPIF | PIM2 |  | ILF3 | PASK | F11R | LSR | OIP5 |  | GINS1 | SORL1 |
|  |  | CSE1L | UTP18 | RAD54L | UPF3B | PPP1R1A |  | PLK1 | BEX2 | MYBL2 | DKC1 | EXOSC2 |  | EPCAM | SLAIN1 |
|  |  | MCM4 | NUP35 | PPIL1 | LIN28 | FZD5 |  | TACC3 | DEPDC1B | AURKA | MPP6 | CEP78 |  | CEP70 | ELAVL2 |
|  |  | FAM169A | CENPN | RCC1 | LMNB1 | IGF2BP1 |  | IPO11 | NUP210 | NASP | KIF2C | TERF1 |  | TFDP2 | PRKCZ |
|  |  | FANCD2 | FEN1 | SIRT1 | WDR33 | PHF5A |  | FKBP5 | SMARCA5 | EEF1E1 | SPAG5 | HMGB3 |  | GPR160 | RHPN2 |
|  |  | TMPO | RFC5 | NARG1 | F2RL1 | SEPHS1 |  | RFWD3 | LYAR | CDC7 | ATP8A1 | MAD2L1 |  | USP28 | OCLN |
|  |  | ORC1L | MYCN | RNF44 | FAM60A |  |  | CDCA7 | USP10 | MAGOH | FANCI | DLGAP5 |  | GRTP1 | ELOVL7 |
|  |  | NUP155 | XPO4 | RUVBL1 | TSR1 |  |  | POLD1 | NUP107 | EIF4E | DTL | ECT2 |  | CGN | PODXL |
|  |  | JARID2 | RBM15 | HEATR1 | RRAGD |  |  | CDT1 | RIF1 | AGPAT5 | SOX13 | PAICS |  | GCH1 | AP1M2 |
|  |  | SEMA4D | CDC20 | PWP2 | CPSF6 |  |  | MSH6 | SFRS1 | SSX2IP | SERBP1 | MSH2 |  | FUBP1 | CLDN7 |
|  |  | CDCA5 | MTHFD1 | HNRNPAB | GEMIN5 |  |  | GINS2 | RG9MTD1 | SLC25A13 | SLC16A1 | SUV39H2 |  | HMMR | LAMA1 |
|  |  | GSG2 | MCM2 | WDHD1 | GLUL |  |  | PAIP2B | SPINT1 | CCNB1 | CDH1 | CHEK1 |  | CNTNAP2 | FUT9 |

**Supplmentary Table S2. Primers for real-time PCR and reverse transcription PCR.**

| Real-time PCR | | | |
| --- | --- | --- | --- |
|  | **Forward** | | **Reverse** |
| c-Myc | CATTCAAGCAGACGAGCA | | CGAGTTAGGTCAGTTTATGCAC |
| Klf4 | CCTTTCAGTGCCAGAAGT | | ACTACGTGGGATTTAAAAGTGC |
| Sox2 | AGGGCTGGACTGCGAACTG | | TTTGCACCCCTCCCAATTC |
| β-actin | GTGCGTGACATCAAAGAGAAGC | | TGGATGCCACAGGATTCCATAC |
| Cntf | GGCTTTCGCAGAGCAATCAC | | GCCAGATAGAGCGGCTACAGA |
| EGF | GAGTCTGCCTGCGGATGGT | | GCTGCAGGGAGGGAGACA |
| OSM | CGGTCCACTACAACACCAGATG | | GCGATGGTATCCCCAGAGAA |
| IL-6 | GGACTGATGCTGGTGAC | | CATTTCTTTGTATCTCTGGAAGTT |
| IL-11 | CATGCCACACCCCAAACAA | | CCCCTCACCCAGGTCTACTG |
| LIF | CCTACCTGCGTCTTACTCCATCA | | TGTTTTCCCCAAAGGCTCAA |
| ItgαV | GACCTTGGAAACCCGATGAA | | TGCTGGTGCACGCTGAAA |
| Itgβ5 | GCTTAGGTTTCGGGTCTTTTGTT | | TCGGTGCCGTGTAGAGAA |
| Oct4 | GAGGCTACAGGGACACCTTTC  (Roche Universal Probes 6) | | GTGCCAAAGTGGGGACCT  (Roche Universal Probes 6) |
| Reverse transcription PCR | | | |
|  | **Forward** | **Reverse** | |
| Oct-4 | CTGAAGCAGAAGAGGATCAC | GGCCGCAGCTTACACATGTT | |
| Nestin | GCTACATACAGGACTCTGCTG | AAACTCTAGACTCACTGGATTCT | |
| Pax6 | TGCCCTTCCATCTTTGCTTG | TCTGCCCGTTCAACATCCTTAG | |
| β-actin | GTGGGGCGCCCCAGGCACCA | CTCCTTAATGTCACGCACGATTTC | |

**Blots/gels**

**
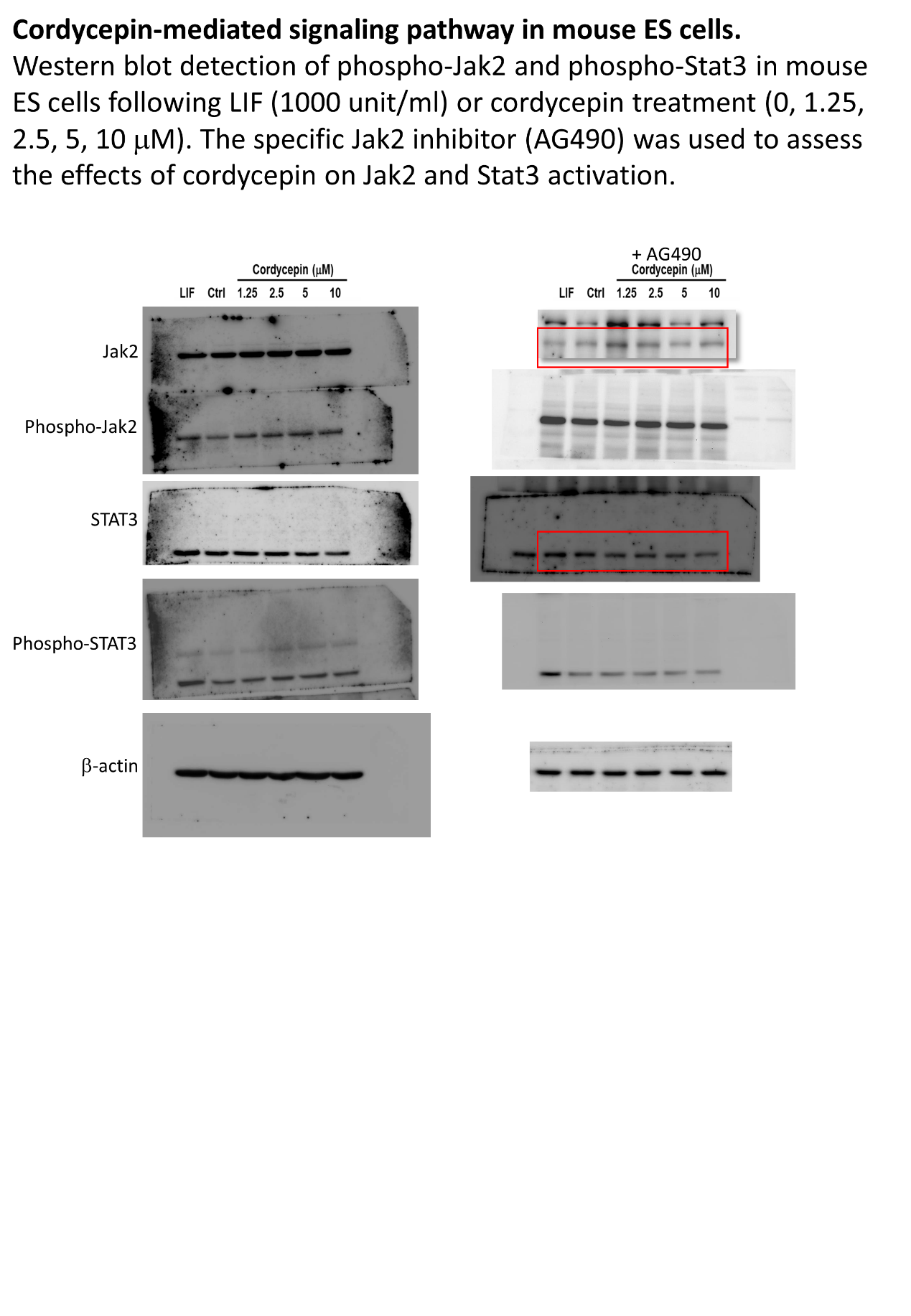
**

**
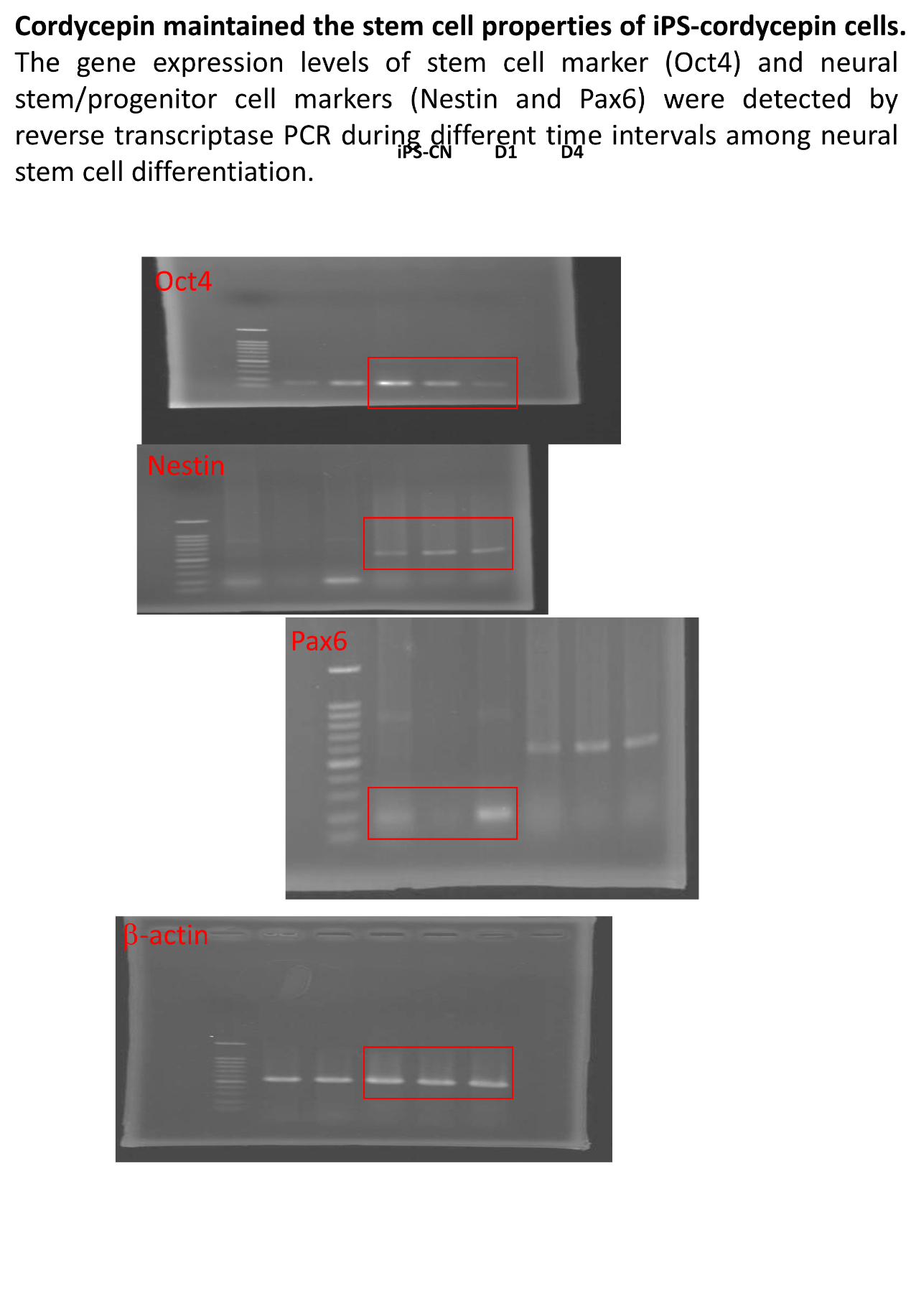
**

**
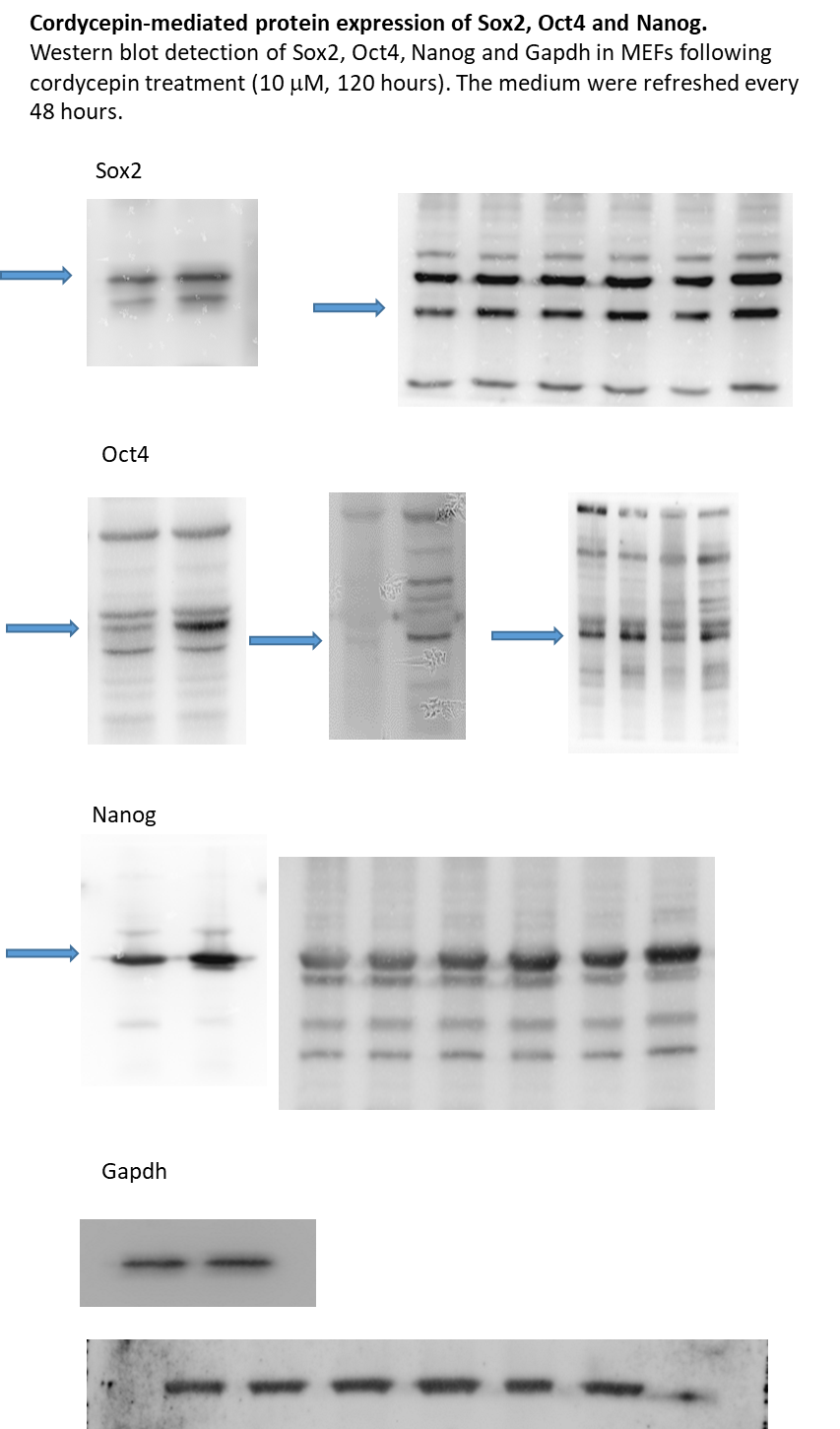
**


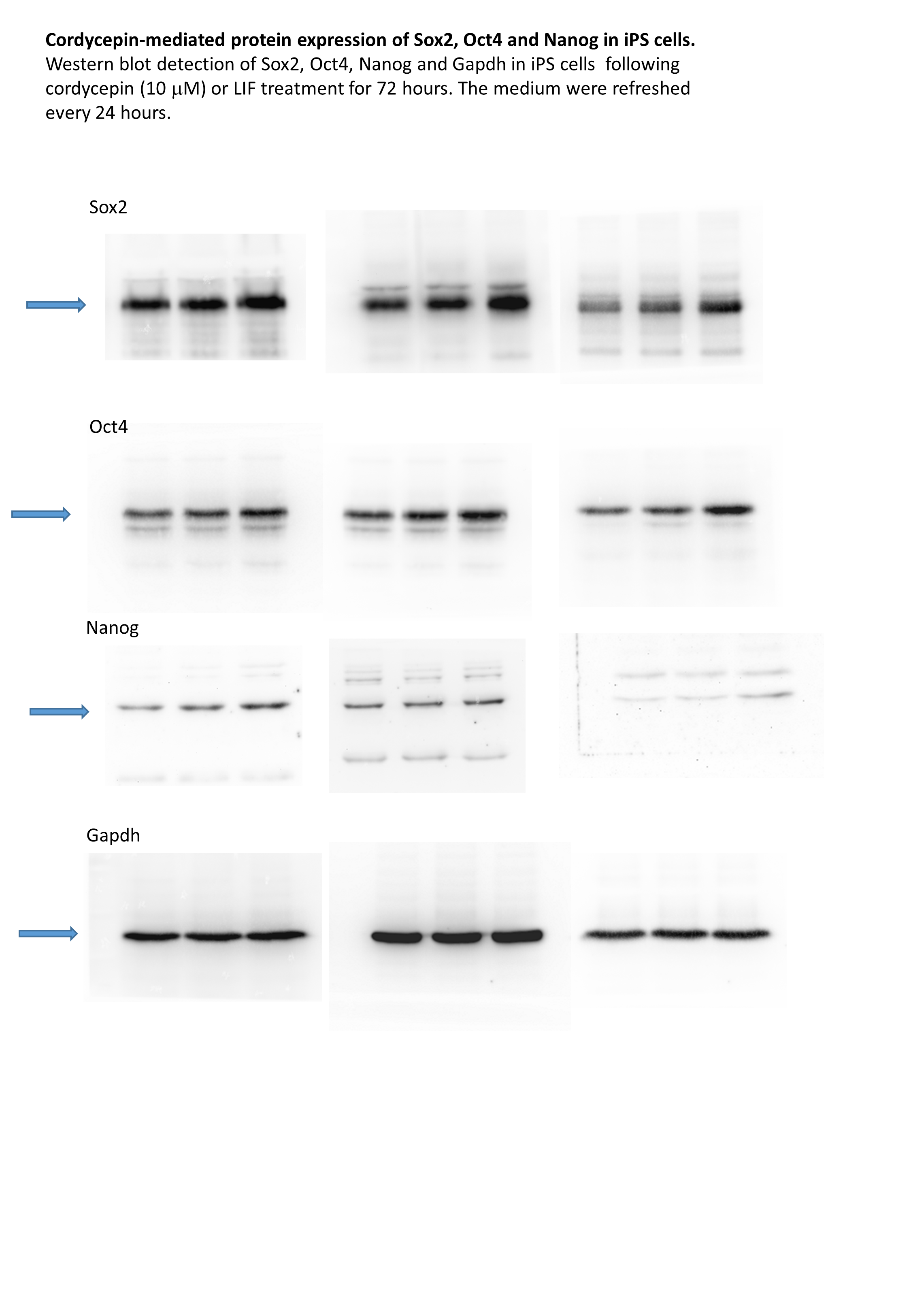

Supplement: Supplementary file 1 — Supplementary information. [file 41598_2020_59154_MOESM1_ESM.docx]
